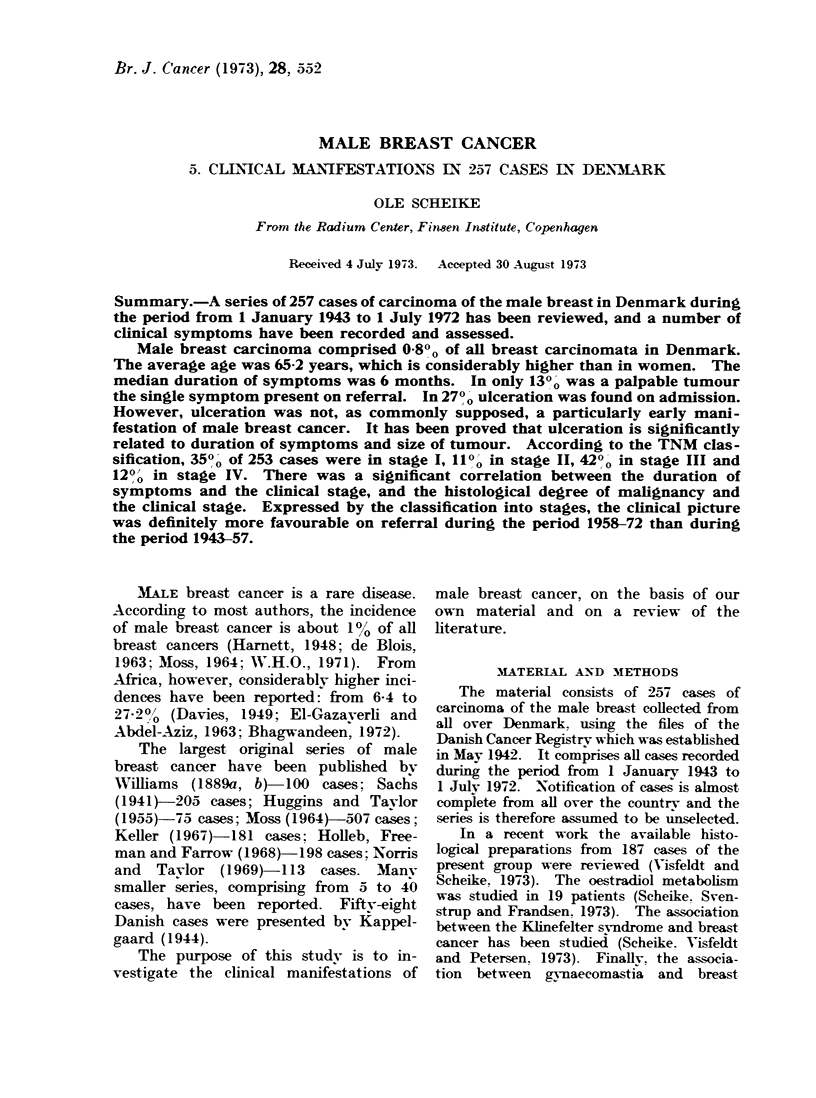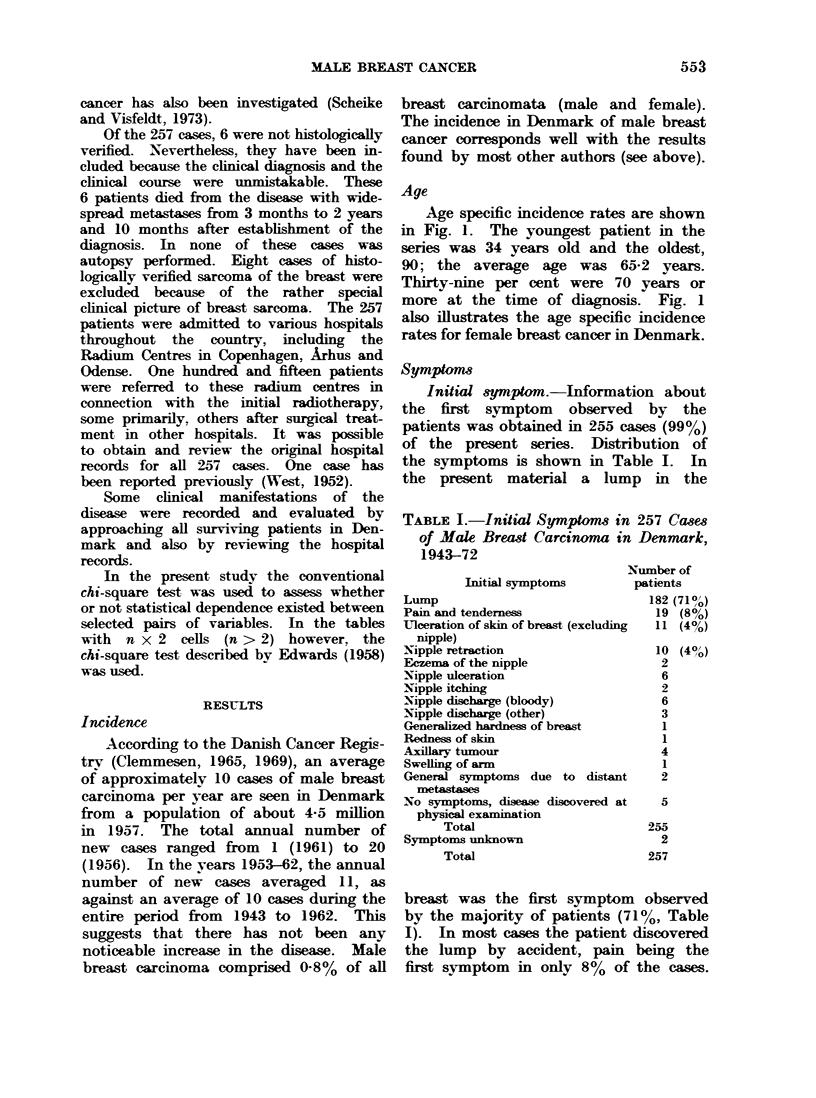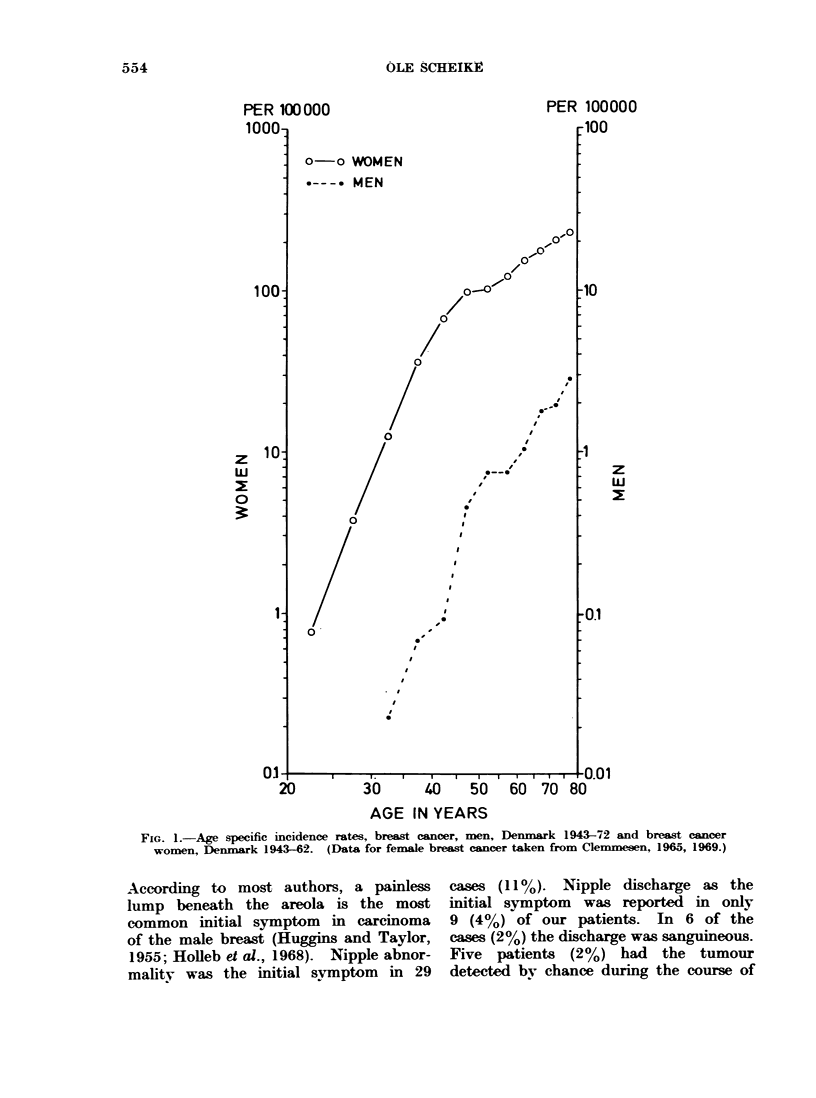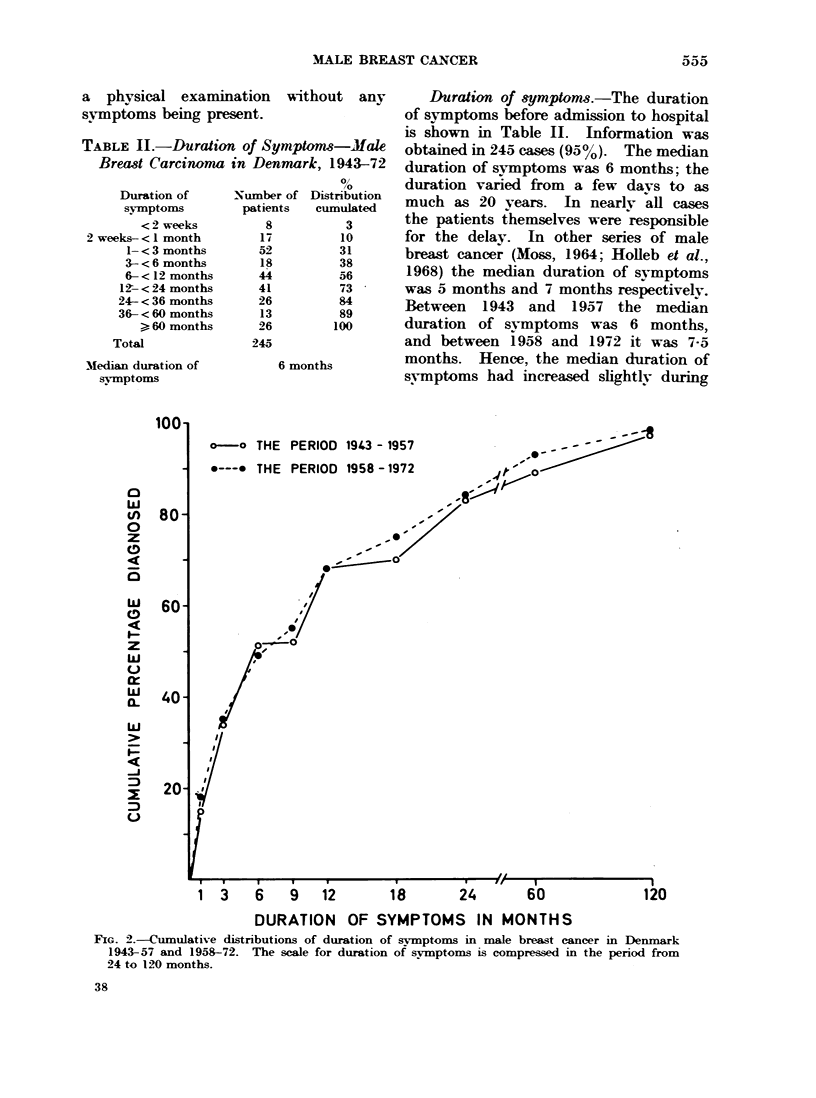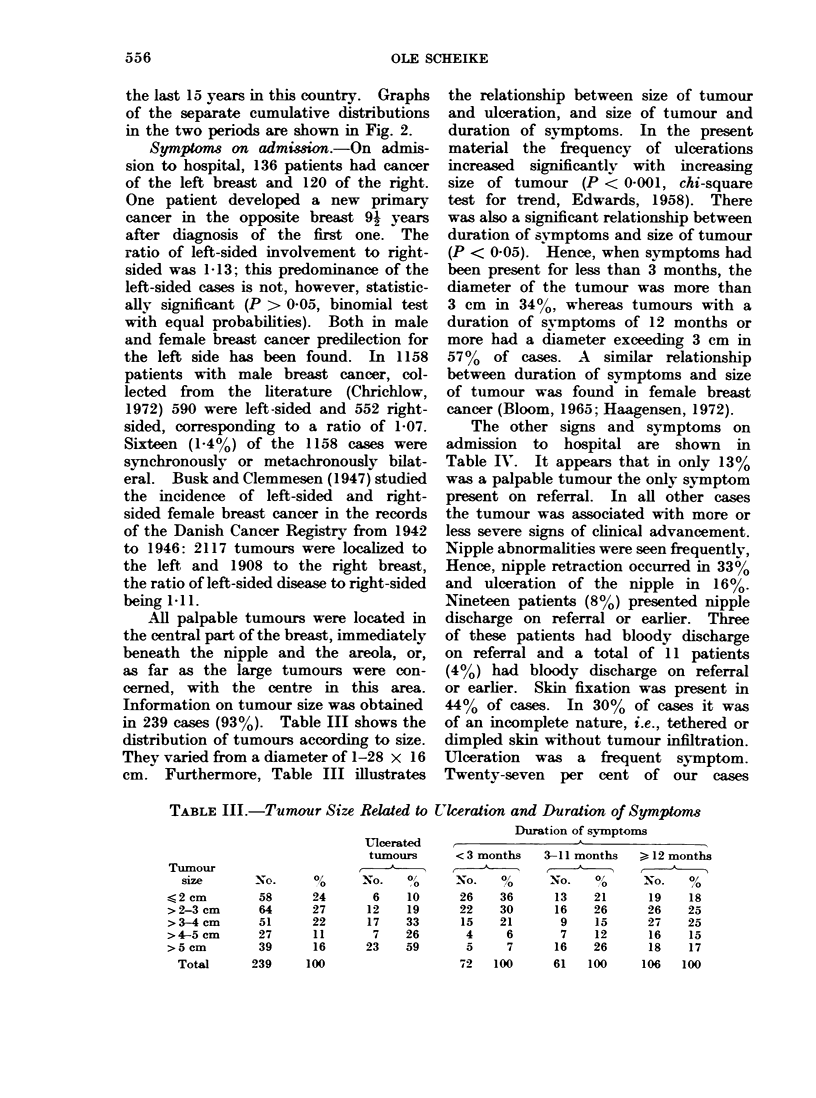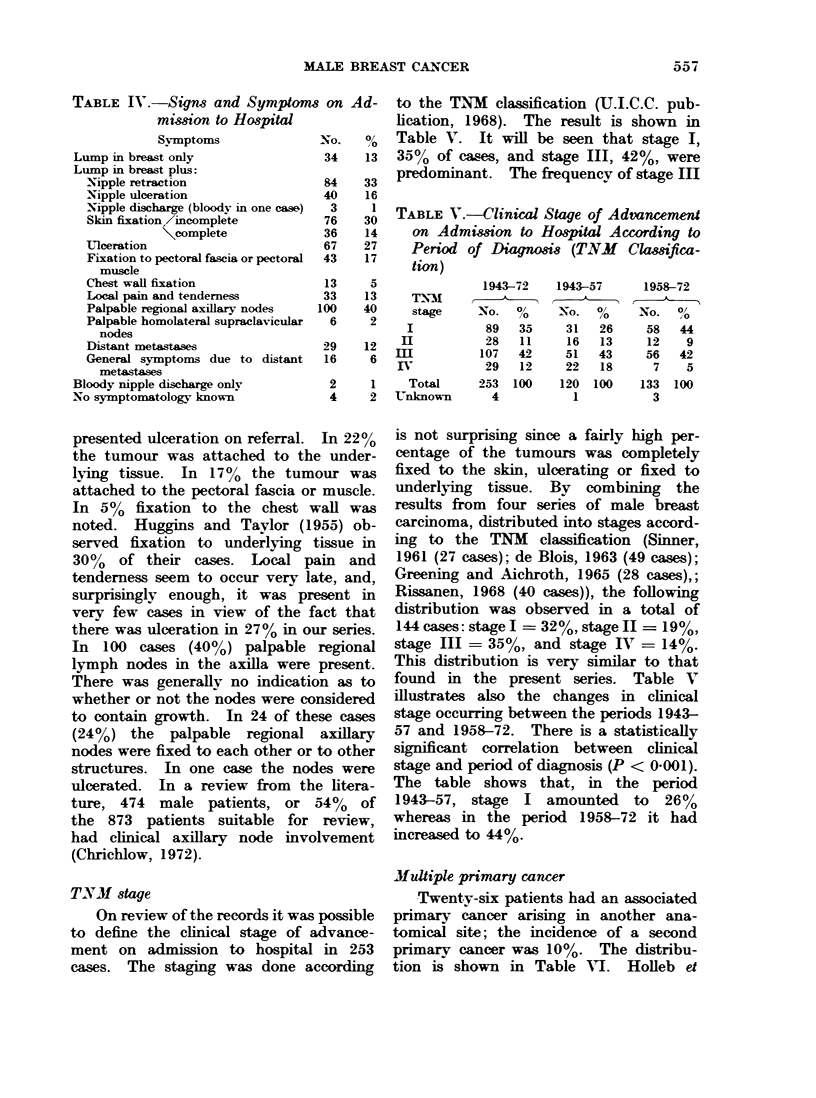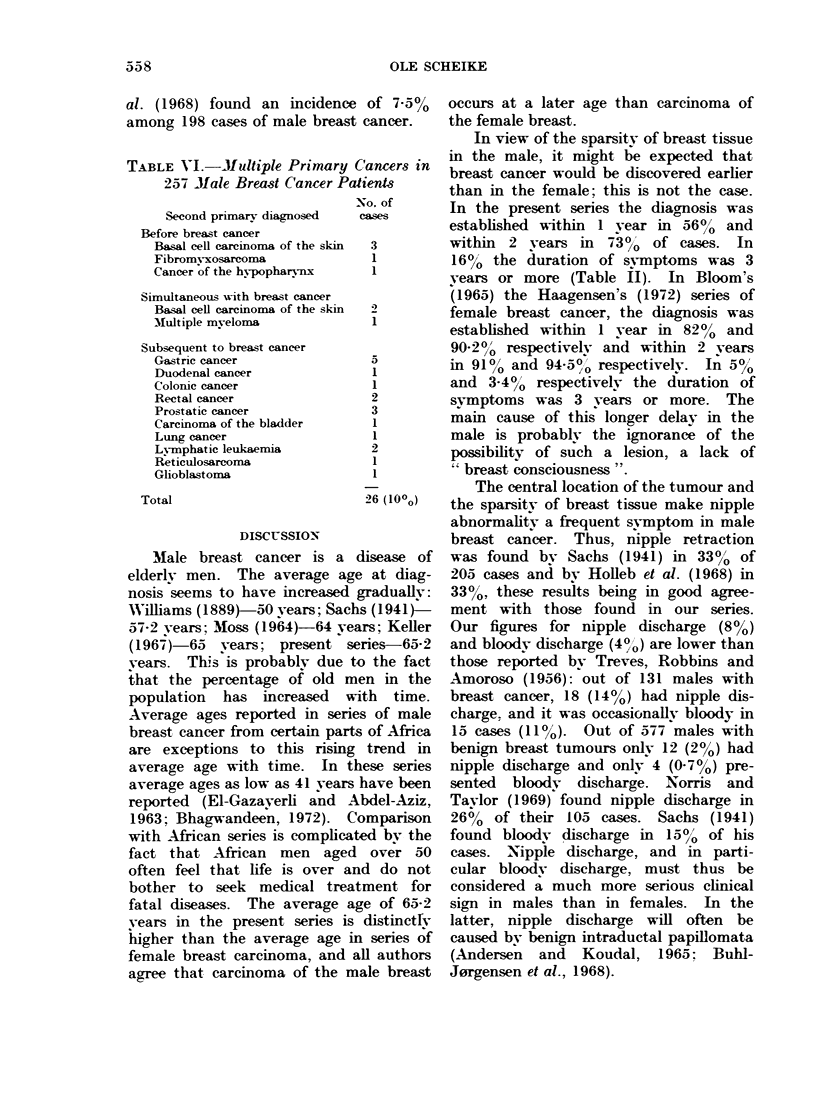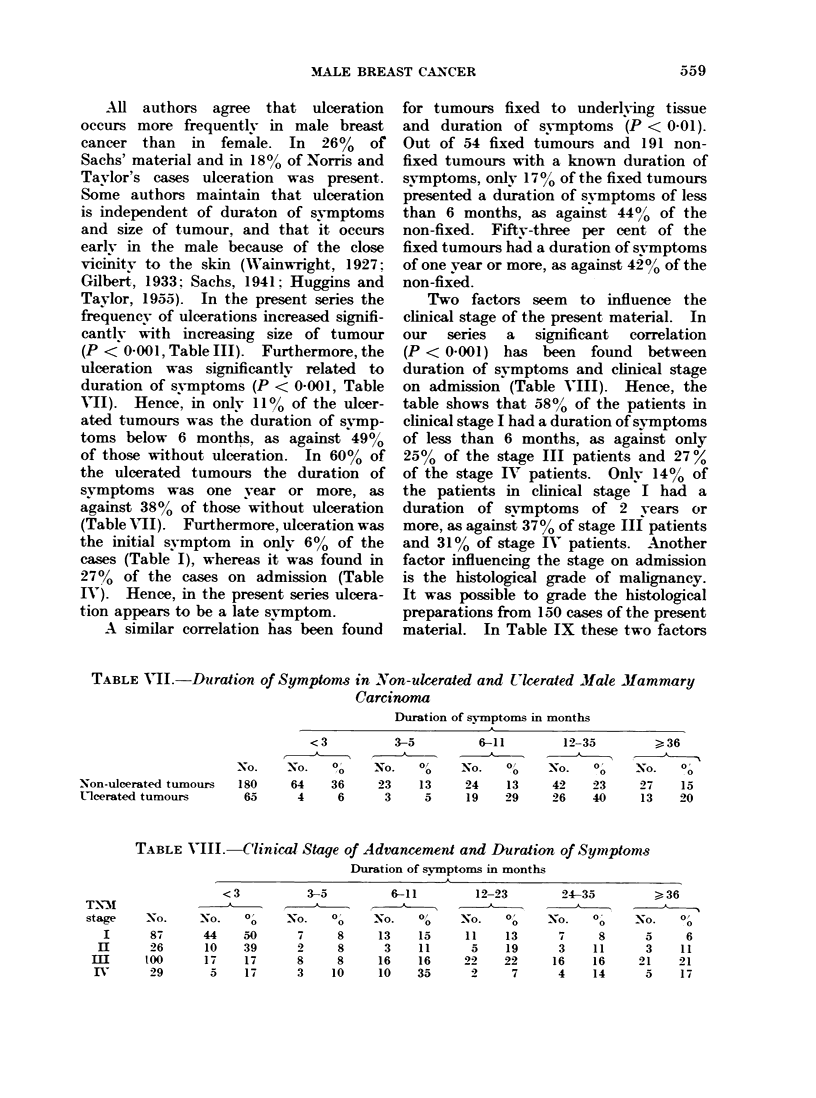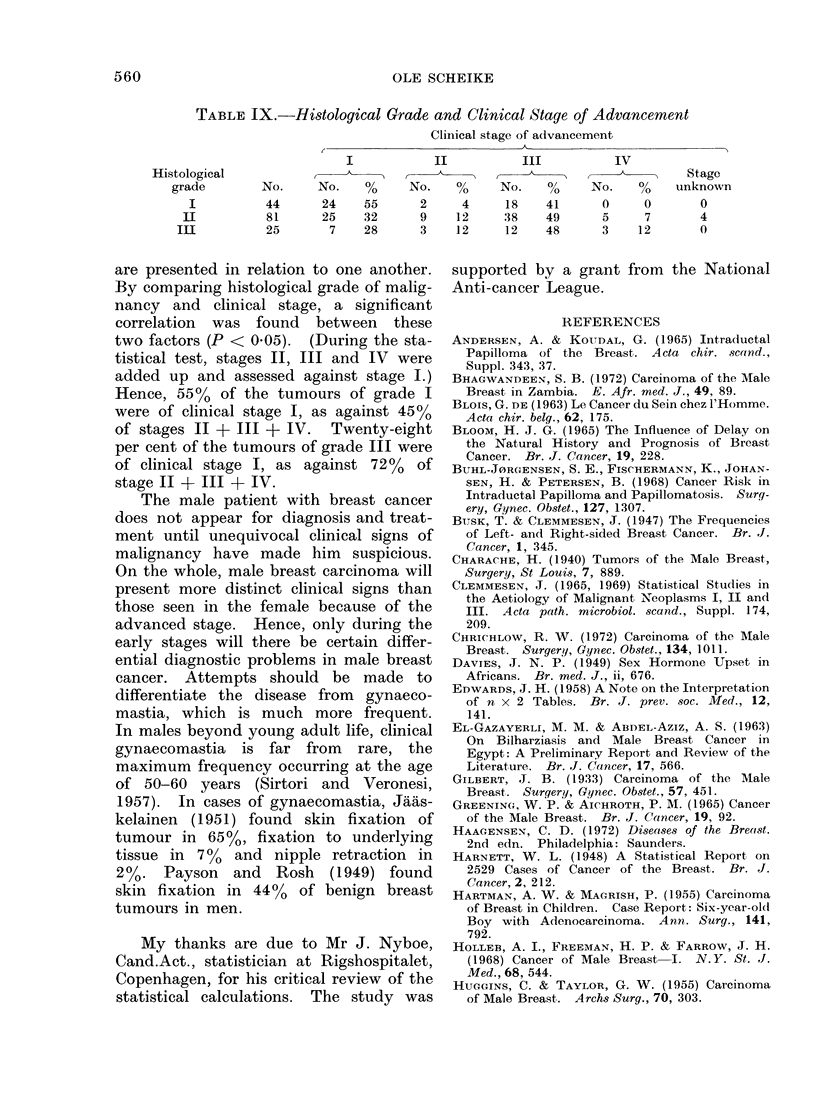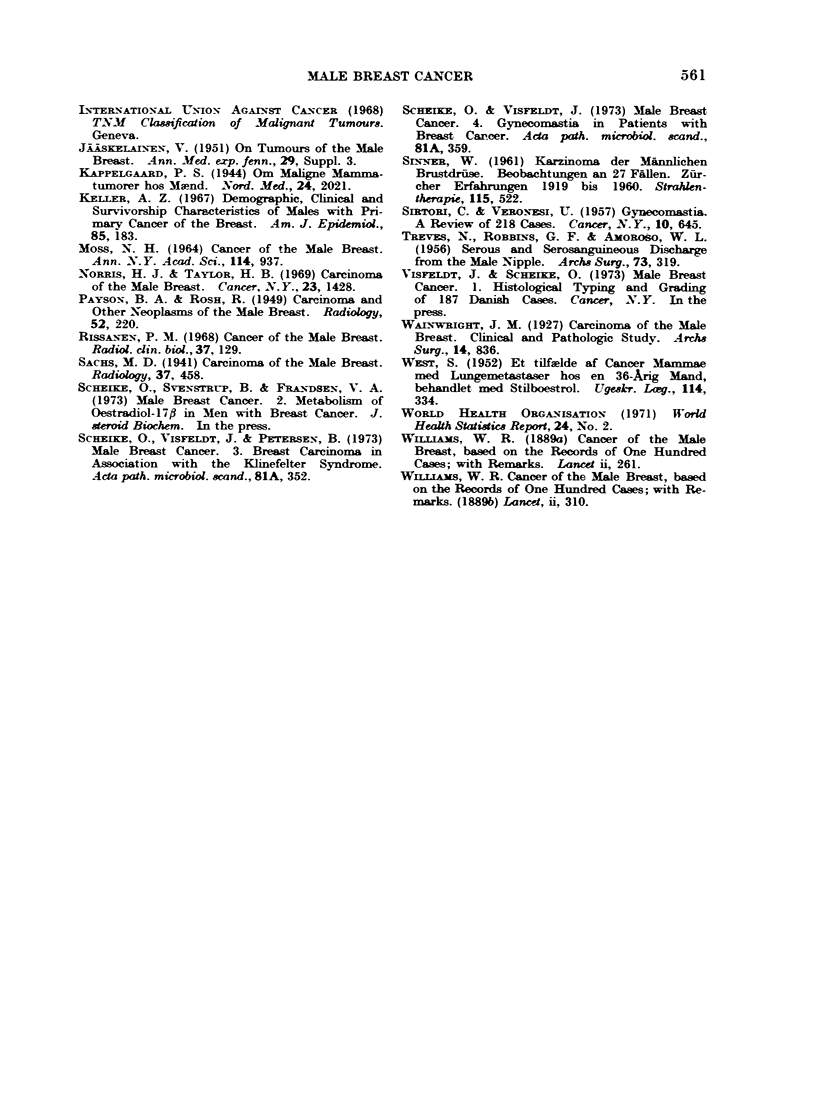# Male Breast Cancer

**DOI:** 10.1038/bjc.1973.185

**Published:** 1973-12

**Authors:** Ole Scheike

## Abstract

A series of 257 cases of carcinoma of the male breast in Denmark during the period from 1 January 1943 to 1 July 1972 has been reviewed, and a number of clinical symptoms have been recorded and assessed.

Male breast carcinoma comprised 0·8% of all breast carcinomata in Denmark. The average age was 65·2 years, which is considerably higher than in women. The median duration of symptoms was 6 months. In only 13% was a palpable tumour the single symptom present on referral. In 27% ulceration was found on admission. However, ulceration was not, as commonly supposed, a particularly early manifestation of male breast cancer. It has been proved that ulceration is significantly related to duration of symptoms and size of tumour. According to the TNM classification, 35% of 253 cases were in stage I, 11% in stage II, 42% in stage III and 12% in stage IV. There was a significant correlation between the duration of symptoms and the clinical stage, and the histological degree of malignancy and the clinical stage. Expressed by the classification into stages, the clinical picture was definitely more favourable on referral during the period 1958-72 than during the period 1943-57.


*Br. J. Cancer* (1973), **28**, 552

### MALE BREAST CANCER

### 5. CLINICAL MANIFESTATIONS IN 257 CASES IN DENMARK

OLE SCHEIKE

*From the Radium Center, Finsen Institute, Copenhagen*

Received 4 July 1973. Accepted 30 August 1973

**Summary.—A series of 257 cases of carcinoma of the male breast in Denmark during the period from 1 January 1943 to 1 July 1972 has been reviewed, and a number of clinical symptoms have been recorded and assessed.**

**Male breast carcinoma comprised 0·8% of all breast carcinomata in Denmark. The average age was 65·2 years, which is considerably higher than in women. The median duration of symptoms was 6 months. In only 13% was a palpable tumour the single symptom present on referral. In 27% ulceration was found on admission. However, ulceration was not, as commonly supposed, a particularly early manifestation of male breast cancer. It has been proved that ulceration is significantly related to duration of symptoms and size of tumour. According to the TNM classification, 35% of 253 cases were in stage I, 11% in stage II, 42% in stage III and 12% in stage IV. There was a significant correlation between the duration of symptoms and the clinical stage, and the histological degree of malignancy and the clinical stage. Expressed by the classification into stages, the clinical picture was definitely more favourable on referral during the period 1958–72 than during the period 1943–57.**

MALE breast cancer is a rare disease. According to most authors, the incidence of male breast cancer is about 1% of all breast cancers (Harnett, 1948; de Blois, 1963; Moss, 1964; W.H.O., 1971). From Africa, however, considerably higher incidences have been reported: from 6·4 to 27·2% (Davies, 1949; El-Gazayerli and Abdel-Aziz, 1963; Bhagwandeen, 1972).

The largest original series of male breast cancer have been published by Williams (1889*a*, *b*)—100 cases; Sachs (1941)—205 cases; Huggins and Taylor (1955)—75 cases; Moss (1964)—507 cases; Keller (1967)—181 cases; Holleb, Freeman and Farrow (1968)—198 cases; Norris and Taylor (1969)—113 cases. Many smaller series, comprising from 5 to 40 cases, have been reported. Fifty-eight Danish cases were presented by Kappelgaard (1944).

The purpose of this study is to investigate the clinical manifestations of male breast cancer, on the basis of our own material and on a review of the literature.

### MATERIAL AND METHODS

The material consists of 257 cases of carcinoma of the male breast collected from all over Denmark, using the files of the Danish Cancer Registry which was established in May 1942. It comprises all cases recorded during the period from 1 January 1943 to 1 July 1972. Notification of cases is almost complete from all over the country and the series is therefore assumed to be unselected.

In a recent work the available histological preparations from 187 cases of the present group were reviewed (Visfeldt and Scheike, 1973). The oestradiol metabolism was studied in 19 patients (Scheike, Svenstrup and Frandsen, 1973). The association between the Klinefelter syndrome and breast cancer has been studied (Scheike, Visfeldt and Petersen, 1973). Finally, the association between gynaecomastia and breast cancer has also been investigated (Scheike and Visfeldt, 1973).

Of the 257 cases, 6 were not histologically verified. Nevertheless, they have been included because the clinical diagnosis and the clinical course were unmistakable. These 6 patients died from the disease with widespread metastases from 3 months to 2 years and 10 months after establishment of the diagnosis. In none of these cases was autopsy performed. Eight cases of histologically verified sarcoma of the breast were excluded because of the rather special clinical picture of breast sarcoma. The 257 patients were admitted to various hospitals throughout the country, including the Radium Centres in Copenhagen, Århus and Odense. One hundred and fifteen patients were referred to these radium centres in connection with the initial radiotherapy, some primarily, others after surgical treatment in other hospitals. It was possible to obtain and review the original hospital records for all 257 cases. One case has been reported previously (West, 1952).

Some clinical manifestations of the disease were recorded and evaluated by approaching all surviving patients in Denmark and also by reviewing the hospital records.

In the present study the conventional *chi*-square test was used to assess whether or not statistical dependence existed between selected pairs of variables. In the tables with $n \times 2$ cells ($n > 2$) however, the *chi*-square test described by Edwards (1958) was used.

### RESULTS

### *Incidence*

According to the Danish Cancer Registry (Clemmesen, 1965, 1969), an average of approximately 10 cases of male breast carcinoma per year are seen in Denmark from a population of about 4·5 million in 1957. The total annual number of new cases ranged from 1 (1961) to 20 (1956). In the years 1953–62, the annual number of new cases averaged 11, as against an average of 10 cases during the entire period from 1943 to 1962. This suggests that there has not been any noticeable increase in the disease. Male breast carcinoma comprised 0·8% of all breast carcinomata (male and female). The incidence in Denmark of male breast cancer corresponds well with the results found by most other authors (see above).

### *Age*

Age specific incidence rates are shown in Fig. 1. The youngest patient in the series was 34 years old and the oldest, 90; the average age was 65·2 years. Thirty-nine per cent were 70 years or more at the time of diagnosis. Fig. 1 also illustrates the age specific incidence rates for female breast cancer in Denmark.

### *Symptoms*

*Initial symptom.*—Information about the first symptom observed by the patients was obtained in 255 cases (99%) of the present series. Distribution of the symptoms is shown in Table I. In the present material a lump in the breast was the first symptom observed by the majority of patients (71%, Table I). In most cases the patient discovered the lump by accident, pain being the first symptom in only 8% of the cases.

TABLE I.—*Initial Symptoms in 257 Cases of Male Breast Carcinoma in Denmark, 1943–72*

| Initial symptoms | Number of patients |
|---|---|
| Lump | 182 (71%) |
| Pain and tenderness | 19 (8%) |
| Ulceration of skin of breast (excluding nipple) | 11 (4%) |
| Nipple retraction | 10 (4%) |
| Eczema of the nipple | 2 |
| Nipple ulceration | 6 |
| Nipple itching | 2 |
| Nipple discharge (bloody) | 6 |
| Nipple discharge (other) | 3 |
| Generalized hardness of breast | 1 |
| Redness of skin | 1 |
| Axillary tumour | 4 |
| Swelling of arm | 1 |
| General symptoms due to distant metastases | 2 |
| No symptoms, disease discovered at physical examination | 5 |
| Total | 255 |
| Symptoms unknown | 2 |
| Total | 257 |

FIG. 1.—Age specific incidence rates, breast cancer, men, Denmark 1943–72 and breast cancer women, Denmark 1943–62. (Data for female breast cancer taken from Clemmesen, 1965, 1969.)

According to most authors, a painless lump beneath the areola is the most common initial symptom in carcinoma of the male breast (Huggins and Taylor, 1955; Holleb *et al.*, 1968). Nipple abnormality was the initial symptom in 29 cases (11%). Nipple discharge as the initial symptom was reported in only 9 (4%) of our patients. In 6 of the cases (2%) the discharge was sanguineous. Five patients (2%) had the tumour detected by chance during the course of a physical examination without any symptoms being present.

TABLE II.—*Duration of Symptoms—Male Breast Carcinoma in Denmark*, 1943–72

| Duration of symptoms | Number of patients | % Distribution cumulated |
|---|---|---|
| < 2 weeks | 8 | 3 |
| 2 weeks– < 1 month | 17 | 10 |
| 1– < 3 months | 52 | 31 |
| 3– < 6 months | 18 | 38 |
| 6– < 12 months | 44 | 56 |
| 12– < 24 months | 41 | 73 |
| 24– < 36 months | 26 | 84 |
| 36– < 60 months | 13 | 89 |
| ⩾ 60 months | 26 | 100 |
| Total | 245 | |
| Median duration of symptoms | 6 months | |

*Duration of symptoms.*—The duration of symptoms before admission to hospital is shown in Table II. Information was obtained in 245 cases (95%). The median duration of symptoms was 6 months; the duration varied from a few days to as much as 20 years. In nearly all cases the patients themselves were responsible for the delay. In other series of male breast cancer (Moss, 1964; Holleb *et al.*, 1968) the median duration of symptoms was 5 months and 7 months respectively. Between 1943 and 1957 the median duration of symptoms was 6 months, and between 1958 and 1972 it was 7·5 months. Hence, the median duration of symptoms had increased slightly during

FIG. 2.—Cumulative distributions of duration of symptoms in male breast cancer in Denmark 1943–57 and 1958–72. The scale for duration of symptoms is compressed in the period from 24 to 120 months.

the last 15 years in this country. Graphs of the separate cumulative distributions in the two periods are shown in Fig. 2.

*Symptoms on admission.*—On admission to hospital, 136 patients had cancer of the left breast and 120 of the right. One patient developed a new primary cancer in the opposite breast 9½ years after diagnosis of the first one. The ratio of left-sided involvement to right-sided was 1·13; this predominance of the left-sided cases is not, however, statistically significant ($P > 0{\cdot}05$, binomial test with equal probabilities). Both in male and female breast cancer predilection for the left side has been found. In 1158 patients with male breast cancer, collected from the literature (Chrichlow, 1972) 590 were left-sided and 552 right-sided, corresponding to a ratio of 1·07. Sixteen (1·4%) of the 1158 cases were synchronously or metachronously bilateral. Busk and Clemmesen (1947) studied the incidence of left-sided and right-sided female breast cancer in the records of the Danish Cancer Registry from 1942 to 1946: 2117 tumours were localized to the left and 1908 to the right breast, the ratio of left-sided disease to right-sided being 1·11.

All palpable tumours were located in the central part of the breast, immediately beneath the nipple and the areola, or, as far as the large tumours were concerned, with the centre in this area. Information on tumour size was obtained in 239 cases (93%). Table III shows the distribution of tumours according to size. They varied from a diameter of 1–28 × 16 cm. Furthermore, Table III illustrates the relationship between size of tumour and ulceration, and size of tumour and duration of symptoms. In the present material the frequency of ulcerations increased significantly with increasing size of tumour ($P < 0{\cdot}001$, *chi*-square test for trend, Edwards, 1958). There was also a significant relationship between duration of symptoms and size of tumour ($P < 0{\cdot}05$). Hence, when symptoms had been present for less than 3 months, the diameter of the tumour was more than 3 cm in 34%, whereas tumours with a duration of symptoms of 12 months or more had a diameter exceeding 3 cm in 57% of cases. A similar relationship between duration of symptoms and size of tumour was found in female breast cancer (Bloom, 1965; Haagensen, 1972).

The other signs and symptoms on admission to hospital are shown in Table IV. It appears that in only 13% was a palpable tumour the only symptom present on referral. In all other cases the tumour was associated with more or less severe signs of clinical advancement. Nipple abnormalities were seen frequently, Hence, nipple retraction occurred in 33% and ulceration of the nipple in 16%. Nineteen patients (8%) presented nipple discharge on referral or earlier. Three of these patients had bloody discharge on referral and a total of 11 patients (4%) had bloody discharge on referral or earlier. Skin fixation was present in 44% of cases. In 30% of cases it was of an incomplete nature, *i.e.*, tethered or dimpled skin without tumour infiltration. Ulceration was a frequent symptom. Twenty-seven per cent of our cases

TABLE III.—*Tumour Size Related to Ulceration and Duration of Symptoms*

| Tumour size | | | Ulcerated tumours | | Duration of symptoms: < 3 months | | 3–11 months | | ⩾ 12 months | |
|---|---|---|---|---|---|---|---|---|---|---|
| | No. | % | No. | % | No. | % | No. | % | No. | % |
| ⩽ 2 cm | 58 | 24 | 6 | 10 | 26 | 36 | 13 | 21 | 19 | 18 |
| > 2–3 cm | 64 | 27 | 12 | 19 | 22 | 30 | 16 | 26 | 26 | 25 |
| > 3–4 cm | 51 | 22 | 17 | 33 | 15 | 21 | 9 | 15 | 27 | 25 |
| > 4–5 cm | 27 | 11 | 7 | 26 | 4 | 6 | 7 | 12 | 16 | 15 |
| > 5 cm | 39 | 16 | 23 | 59 | 5 | 7 | 16 | 26 | 18 | 17 |
| Total | 239 | 100 | | | 72 | 100 | 61 | 100 | 106 | 100 |

TABLE IV.—*Signs and Symptoms on Admission to Hospital*

| Symptoms | No. | % |
|---|---|---|
| Lump in breast only | 34 | 13 |
| Lump in breast plus: | | |
| Nipple retraction | 84 | 33 |
| Nipple ulceration | 40 | 16 |
| Nipple discharge (bloody in one case) | 3 | 1 |
| Skin fixation / incomplete | 76 | 30 |
| Skin fixation \ complete | 36 | 14 |
| Ulceration | 67 | 27 |
| Fixation to pectoral fascia or pectoral muscle | 43 | 17 |
| Chest wall fixation | 13 | 5 |
| Local pain and tenderness | 33 | 13 |
| Palpable regional axillary nodes | 100 | 40 |
| Palpable homolateral supraclavicular nodes | 6 | 2 |
| Distant metastases | 29 | 12 |
| General symptoms due to distant metastases | 16 | 6 |
| Bloody nipple discharge only | 2 | 1 |
| No symptomatology known | 4 | 2 |

presented ulceration on referral. In 22% the tumour was attached to the underlying tissue. In 17% the tumour was attached to the pectoral fascia or muscle. In 5% fixation to the chest wall was noted. Huggins and Taylor (1955) observed fixation to underlying tissue in 30% of their cases. Local pain and tenderness seem to occur very late, and, surprisingly enough, it was present in very few cases in view of the fact that there was ulceration in 27% in our series. In 100 cases (40%) palpable regional lymph nodes in the axilla were present. There was generally no indication as to whether or not the nodes were considered to contain growth. In 24 of these cases (24%) the palpable regional axillary nodes were fixed to each other or to other structures. In one case the nodes were ulcerated. In a review from the literature, 474 male patients, or 54% of the 873 patients suitable for review, had clinical axillary node involvement (Chrichlow, 1972).

### *TNM stage*

On review of the records it was possible to define the clinical stage of advancement on admission to hospital in 253 cases. The staging was done according to the TNM classification (U.I.C.C. publication, 1968). The result is shown in Table V. It will be seen that stage I, 35% of cases, and stage III, 42%, were predominant. The frequency of stage III

TABLE V.—*Clinical Stage of Advancement on Admission to Hospital According to Period of Diagnosis (TNM Classification)*

| TNM stage | 1943–72 No. | 1943–72 % | 1943–57 No. | 1943–57 % | 1958–72 No. | 1958–72 % |
|---|---|---|---|---|---|---|
| I | 89 | 35 | 31 | 26 | 58 | 44 |
| II | 28 | 11 | 16 | 13 | 12 | 9 |
| III | 107 | 42 | 51 | 43 | 56 | 42 |
| IV | 29 | 12 | 22 | 18 | 7 | 5 |
| Total | 253 | 100 | 120 | 100 | 133 | 100 |
| Unknown | 4 | | 1 | | 3 | |

is not surprising since a fairly high percentage of the tumours was completely fixed to the skin, ulcerating or fixed to underlying tissue. By combining the results from four series of male breast carcinoma, distributed into stages according to the TNM classification (Sinner, 1961 (27 cases); de Blois, 1963 (49 cases); Greening and Aichroth, 1965 (28 cases),; Rissanen, 1968 (40 cases)), the following distribution was observed in a total of 144 cases: stage I = 32%, stage II = 19%, stage III = 35%, and stage IV = 14%. This distribution is very similar to that found in the present series. Table V illustrates also the changes in clinical stage occurring between the periods 1943–57 and 1958–72. There is a statistically significant correlation between clinical stage and period of diagnosis ($P < 0{\cdot}001$). The table shows that, in the period 1943–57, stage I amounted to 26% whereas in the period 1958–72 it had increased to 44%.

### *Multiple primary cancer*

Twenty-six patients had an associated primary cancer arising in another anatomical site; the incidence of a second primary cancer was 10%. The distribution is shown in Table VI. Holleb *et*

*al.* (1968) found an incidence of 7·5% among 198 cases of male breast cancer.

TABLE VI.—*Multiple Primary Cancers in 257 Male Breast Cancer Patients*

| Second primary diagnosed | No. of cases |
|---|---|
| Before breast cancer | |
| Basal cell carcinoma of the skin | 3 |
| Fibromyxosarcoma | 1 |
| Cancer of the hypopharynx | 1 |
| Simultaneous with breast cancer | |
| Basal cell carcinoma of the skin | 2 |
| Multiple myeloma | 1 |
| Subsequent to breast cancer | |
| Gastric cancer | 5 |
| Duodenal cancer | 1 |
| Colonic cancer | 1 |
| Rectal cancer | 2 |
| Prostatic cancer | 3 |
| Carcinoma of the bladder | 1 |
| Lung cancer | 1 |
| Lymphatic leukaemia | 2 |
| Reticulosarcoma | 1 |
| Glioblastoma | 1 |
| Total | 26 (10%) |

### DISCUSSION

Male breast cancer is a disease of elderly men. The average age at diagnosis seems to have increased gradually: Williams (1889)—50 years; Sachs (1941)—57·2 years; Moss (1964)—64 years; Keller (1967)—65 years; present series—65·2 years. This is probably due to the fact that the percentage of old men in the population has increased with time. Average ages reported in series of male breast cancer from certain parts of Africa are exceptions to this rising trend in average age with time. In these series average ages as low as 41 years have been reported (El-Gazayerli and Abdel-Aziz, 1963; Bhagwandeen, 1972). Comparison with African series is complicated by the fact that African men aged over 50 often feel that life is over and do not bother to seek medical treatment for fatal diseases. The average age of 65·2 years in the present series is distinctly higher than the average age in series of female breast carcinoma, and all authors agree that carcinoma of the male breast occurs at a later age than carcinoma of the female breast.

In view of the sparsity of breast tissue in the male, it might be expected that breast cancer would be discovered earlier than in the female; this is not the case. In the present series the diagnosis was established within 1 year in 56% and within 2 years in 73% of cases. In 16% the duration of symptoms was 3 years or more (Table II). In Bloom's (1965) the Haagensen's (1972) series of female breast cancer, the diagnosis was established within 1 year in 82% and 90·2% respectively and within 2 years in 91% and 94·5% respectively. In 5% and 3·4% respectively the duration of symptoms was 3 years or more. The main cause of this longer delay in the male is probably the ignorance of the possibility of such a lesion, a lack of " breast consciousness ".

The central location of the tumour and the sparsity of breast tissue make nipple abnormality a frequent symptom in male breast cancer. Thus, nipple retraction was found by Sachs (1941) in 33% of 205 cases and by Holleb *et al.* (1968) in 33%, these results being in good agreement with those found in our series. Our figures for nipple discharge (8%) and bloody discharge (4%) are lower than those reported by Treves, Robbins and Amoroso (1956): out of 131 males with breast cancer, 18 (14%) had nipple discharge, and it was occasionally bloody in 15 cases (11%). Out of 577 males with benign breast tumours only 12 (2%) had nipple discharge and only 4 (0·7%) presented bloody discharge. Norris and Taylor (1969) found nipple discharge in 26% of their 105 cases. Sachs (1941) found bloody discharge in 15% of his cases. Nipple discharge, and in particular bloody discharge, must thus be considered a much more serious clinical sign in males than in females. In the latter, nipple discharge will often be caused by benign intraductal papillomata (Andersen and Koudal, 1965; Buhl-Jørgensen *et al.*, 1968).

All authors agree that ulceration occurs more frequently in male breast cancer than in female. In 26% of Sachs' material and in 18% of Norris and Taylor's cases ulceration was present. Some authors maintain that ulceration is independent of duraton of symptoms and size of tumour, and that it occurs early in the male because of the close vicinity to the skin (Wainwright, 1927; Gilbert, 1933; Sachs, 1941; Huggins and Taylor, 1955). In the present series the frequency of ulcerations increased significantly with increasing size of tumour ($P < 0{\cdot}001$, Table III). Furthermore, the ulceration was significantly related to duration of symptoms ($P < 0{\cdot}001$, Table VII). Hence, in only 11% of the ulcerated tumours was the duration of symptoms below 6 months, as against 49% of those without ulceration. In 60% of the ulcerated tumours the duration of symptoms was one year or more, as against 38% of those without ulceration (Table VII). Furthermore, ulceration was the initial symptom in only 6% of the cases (Table I), whereas it was found in 27% of the cases on admission (Table IV). Hence, in the present series ulceration appears to be a late symptom.

A similar correlation has been found for tumours fixed to underlying tissue and duration of symptoms ($P < 0{\cdot}01$). Out of 54 fixed tumours and 191 non-fixed tumours with a known duration of symptoms, only 17% of the fixed tumours presented a duration of symptoms of less than 6 months, as against 44% of the non-fixed. Fifty-three per cent of the fixed tumours had a duration of symptoms of one year or more, as against 42% of the non-fixed.

Two factors seem to influence the clinical stage of the present material. In our series a significant correlation ($P < 0{\cdot}001$) has been found between duration of symptoms and clinical stage on admission (Table VIII). Hence, the table shows that 58% of the patients in clinical stage I had a duration of symptoms of less than 6 months, as against only 25% of the stage III patients and 27% of the stage IV patients. Only 14% of the patients in clinical stage I had a duration of symptoms of 2 years or more, as against 37% of stage III patients and 31% of stage IV patients. Another factor influencing the stage on admission is the histological grade of malignancy. It was possible to grade the histological preparations from 150 cases of the present material. In Table IX these two factors

TABLE VII.—*Duration of Symptoms in Non-ulcerated and Ulcerated Male Mammary Carcinoma*

| | | Duration of symptoms in months | | | | | | | | | |
|---|---|---|---|---|---|---|---|---|---|---|---|
| | | <3 | | 3–5 | | 6–11 | | 12–35 | | ≥36 | |
| | No. | No. | % | No. | % | No. | % | No. | % | No. | % |
| Non-ulcerated tumours | 180 | 64 | 36 | 23 | 13 | 24 | 13 | 42 | 23 | 27 | 15 |
| Ulcerated tumours | 65 | 4 | 6 | 3 | 5 | 19 | 29 | 26 | 40 | 13 | 20 |

TABLE VIII.—*Clinical Stage of Advancement and Duration of Symptoms*

| | | Duration of symptoms in months | | | | | | | | | | | |
|---|---|---|---|---|---|---|---|---|---|---|---|---|---|
| TNM | | <3 | | 3–5 | | 6–11 | | 12–23 | | 24–35 | | ≥36 | |
| stage | No. | No. | % | No. | % | No. | % | No. | % | No. | % | No. | % |
| I | 87 | 44 | 50 | 7 | 8 | 13 | 15 | 11 | 13 | 7 | 8 | 5 | 6 |
| II | 26 | 10 | 39 | 2 | 8 | 3 | 11 | 5 | 19 | 3 | 11 | 3 | 11 |
| III | 100 | 17 | 17 | 8 | 8 | 16 | 16 | 22 | 22 | 16 | 16 | 21 | 21 |
| IV | 29 | 5 | 17 | 3 | 10 | 10 | 35 | 2 | 7 | 4 | 14 | 5 | 17 |

TABLE IX.—*Histological Grade and Clinical Stage of Advancement*

| Histological grade | No. | Clinical stage of advancement: I No. | I % | II No. | II % | III No. | III % | IV No. | IV % | Stage unknown |
|---|---|---|---|---|---|---|---|---|---|---|
| I | 44 | 24 | 55 | 2 | 4 | 18 | 41 | 0 | 0 | 0 |
| II | 81 | 25 | 32 | 9 | 12 | 38 | 49 | 5 | 7 | 4 |
| III | 25 | 7 | 28 | 3 | 12 | 12 | 48 | 3 | 12 | 0 |

are presented in relation to one another. By comparing histological grade of malignancy and clinical stage, a significant correlation was found between these two factors ($P < 0{\cdot}05$). (During the statistical test, stages II, III and IV were added up and assessed against stage I.) Hence, 55% of the tumours of grade I were of clinical stage I, as against 45% of stages II + III + IV. Twenty-eight per cent of the tumours of grade III were of clinical stage I, as against 72% of stage II + III + IV.

The male patient with breast cancer does not appear for diagnosis and treatment until unequivocal clinical signs of malignancy have made him suspicious. On the whole, male breast carcinoma will present more distinct clinical signs than those seen in the female because of the advanced stage. Hence, only during the early stages will there be certain differential diagnostic problems in male breast cancer. Attempts should be made to differentiate the disease from gynaecomastia, which is much more frequent. In males beyond young adult life, clinical gynaecomastia is far from rare, the maximum frequency occurring at the age of 50–60 years (Sirtori and Veronesi, 1957). In cases of gynaecomastia, Jääskelainen (1951) found skin fixation of tumour in 65%, fixation to underlying tissue in 7% and nipple retraction in 2%. Payson and Rosh (1949) found skin fixation in 44% of benign breast tumours in men.

My thanks are due to Mr J. Nyboe, Cand.Act., statistician at Rigshospitalet, Copenhagen, for his critical review of the statistical calculations. The study was supported by a grant from the National Anti-cancer League.

### REFERENCES

ANDERSEN, A. & KOUDAL, G. (1965) Intraductal Papilloma of the Breast. *Acta chir. scand.*, Suppl. 343, 37.
BHAGWANDEEN, S. B. (1972) Carcinoma of the Male Breast in Zambia. *E. Afr. med. J.*, **49**, 89.
BLOIS, G. DE (1963) Le Cancer du Sein chez l'Homme. *Acta chir. belg.*, **62**, 175.
BLOOM, H. J. G. (1965) The Influence of Delay on the Natural History and Prognosis of Breast Cancer. *Br. J. Cancer*, **19**, 228.
BUHL-JØRGENSEN, S. E., FISCHERMANN, K., JOHANSEN, H. & PETERSEN, B. (1968) Cancer Risk in Intraductal Papilloma and Papillomatosis. *Surgery, Gynec. Obstet.*, **127**, 1307.
BUSK, T. & CLEMMESEN, J. (1947) The Frequencies of Left- and Right-sided Breast Cancer. *Br. J. Cancer*, **1**, 345.
CHARACHE, H. (1940) Tumors of the Male Breast, *Surgery, St Louis*, **7**, 889.
CLEMMESEN, J. (1965, 1969) Statistical Studies in the Aetiology of Malignant Neoplasms I, II and III. *Acta path. microbiol. scand.*, Suppl. 174, 209.
CHRICHLOW, R. W. (1972) Carcinoma of the Male Breast. *Surgery, Gynec. Obstet.*, **134**, 1011.
DAVIES, J. N. P. (1949) Sex Hormone Upset in Africans. *Br. med. J.*, ii, 676.
EDWARDS, J. H. (1958) A Note on the Interpretation of $n \times 2$ Tables. *Br. J. prev. soc. Med.*, **12**, 141.
EL-GAZAYERLI, M. M. & ABDEL-AZIZ, A. S. (1963) On Bilharziasis and Male Breast Cancer in Egypt: A Preliminary Report and Review of the Literature. *Br. J. Cancer*, **17**, 566.
GILBERT, J. B. (1933) Carcinoma of the Male Breast. *Surgery, Gynec. Obstet.*, **57**, 451.
GREENING, W. P. & AICHROTH, P. M. (1965) Cancer of the Male Breast. *Br. J. Cancer*, **19**, 92.
HAAGENSEN, C. D. (1972) *Diseases of the Breast.* 2nd edn. Philadelphia: Saunders.
HARNETT, W. L. (1948) A Statistical Report on 2529 Cases of Cancer of the Breast. *Br. J. Cancer*, **2**, 212.
HARTMAN, A. W. & MAGRISH, P. (1955) Carcinoma of Breast in Children. Case Report: Six-year-old Boy with Adenocarcinoma. *Ann. Surg.*, **141**, 792.
HOLLEB, A. I., FREEMAN, H. P. & FARROW, J. H. (1968) Cancer of Male Breast—I. *N.Y. St. J. Med.*, **68**, 544.
HUGGINS, C. & TAYLOR, G. W. (1955) Carcinoma of Male Breast. *Archs Surg.*, **70**, 303.

INTERNATIONAL UNION AGAINST CANCER (1968) *TNM Classification of Malignant Tumours.* Geneva.

JÄÄSKELAINEN, V. (1951) On Tumours of the Male Breast. *Ann. Med. exp. fenn.*, **29**, Suppl. 3.

KAPPELGAARD, P. S. (1944) Om Maligne Mammatumorer hos Mænd. *Nord. Med.*, **24**, 2021.

KELLER, A. Z. (1967) Demographic, Clinical and Survivorship Characteristics of Males with Primary Cancer of the Breast. *Am. J. Epidemiol.*, **85**, 183.

MOSS, N. H. (1964) Cancer of the Male Breast. *Ann. N.Y. Acad. Sci.*, **114**, 937.

NORRIS, H. J. & TAYLOR, H. B. (1969) Carcinoma of the Male Breast. *Cancer, N.Y.*, **23**, 1428.

PAYSON, B. A. & ROSH, R. (1949) Carcinoma and Other Neoplasms of the Male Breast. *Radiology*, **52**, 220.

RISSANEN, P. M. (1968) Cancer of the Male Breast. *Radiol. clin. biol.*, **37**, 129.

SACHS, M. D. (1941) Carcinoma of the Male Breast. *Radiology*, **37**, 458.

SCHEIKE, O., SVENSTRUP, B. & FRANDSEN, V. A. (1973) Male Breast Cancer. 2. Metabolism of Oestradiol-17$\beta$ in Men with Breast Cancer. *J. steroid Biochem.* In the press.

SCHEIKE, O., VISFELDT, J. & PETERSEN, B. (1973) Male Breast Cancer. 3. Breast Carcinoma in Association with the Klinefelter Syndrome. *Acta path. microbiol. scand.*, **81A**, 352.

SCHEIKE, O. & VISFELDT, J. (1973) Male Breast Cancer. 4. Gynecomastia in Patients with Breast Cancer. *Acta path. microbiol. scand.*, **81A**, 359.

SINNER, W. (1961) Karzinoma der Männlichen Brustdrüse. Beobachtungen an 27 Fällen. Zürcher Erfahrungen 1919 bis 1960. *Strahlentherapie*, **115**, 522.

SIRTORI, C. & VERONESI, U. (1957) Gynecomastia. A Review of 218 Cases. *Cancer, N.Y.*, **10**, 645.

TREVES, N., ROBBINS, G. F. & AMOROSO, W. L. (1956) Serous and Serosanguineous Discharge from the Male Nipple. *Archs Surg.*, **73**, 319.

VISFELDT, J. & SCHEIKE, O. (1973) Male Breast Cancer. 1. Histological Typing and Grading of 187 Danish Cases. *Cancer, N.Y.* In the press.

WAINWRIGHT, J. M. (1927) Carcinoma of the Male Breast. Clinical and Pathologic Study. *Archs Surg.*, **14**, 836.

WEST, S. (1952) Et tilfælde af Cancer Mammae med Lungemetastaser hos en 36-Årig Mand, behandlet med Stilboestrol. *Ugeskr. Læg.*, **114**, 334.

WORLD HEALTH ORGANISATION (1971) *World Health Statistics Report*, **24**, No. 2.

WILLIAMS, W. R. (1889*a*) Cancer of the Male Breast, based on the Records of One Hundred Cases; with Remarks. *Lancet* ii, 261.

WILLIAMS, W. R. Cancer of the Male Breast, based on the Records of One Hundred Cases; with Remarks. (1889*b*) *Lancet*, ii, 310.